# ApoC3 Attenuates Platelet Activation Through GPIIb/IIIa Receptor Interaction

**DOI:** 10.3390/cells14181411

**Published:** 2025-09-09

**Authors:** Michael Holzer, Eva Gruden, Sanja Curcic, Gerhard Cvirn, Gunther Marsche

**Affiliations:** 1 Otto-Loewi Research Center, Division of Pharmacology, Medical University of Graz, 8010 Graz, Austria; michael.holzer@medunigraz.at (M.H.); eva.knuplez@medunigraz.at (E.G.); 2 BioTechMed-Graz, 8010 Graz, Austria; 3 Division of Haematology, Medical University of Graz, 8010 Graz, Austria; 4 Division of Medical Physics and Biophysics, Gottfried Schatz Research Center, Medical University of Graz, 8010 Graz, Austria; sanja.curcic@medunigraz.at; 5 Division of Medicinal Chemistry, Otto-Loewi Research Center, Medical University of Graz, 8010 Graz, Austria; gerhard.cvirn@medunigraz.at

**Keywords:** apolipoproteins, platelets, lipoprotein/receptors, GPIIb/IIIa, vascular biology, apoC3

## Abstract

Apolipoprotein C3 (apoC3) is a key regulator of triglyceride metabolism and has emerged as a potential therapeutic target for reducing the risk of cardiovascular disease. However, its broader physiological functions are not fully understood. This study investigates the role of apoC3 in platelet function and thrombus formation. Interestingly, human apoC3 was found to rapidly inhibit platelet activation over the tested concentration range of 0.1–10 µg/mL, with significant effects observed at low concentrations and brief pre-incubation times (from 1 min). At a concentration of 10 µg/mL, apoC3 suppressed platelet activation by approximately 70% in response to ADP and by approximately 40% in response to collagen stimulation. Depleting apoC3 from human serum enhanced platelet aggregation by more than 25 % (1.28 ± 0.19 vs. vehicle), indicating an endogenous regulatory function of apoC3. Mechanistically, apoC3 binding to platelets reduced both GPIIb/IIIa activation and P-selectin expression by around 20%. ApoC3 binding to platelets increased when platelets were activated by ADP and was partially mediated by GPIIb/IIIa, implicating this integrin as a functionally relevant receptor. Taken together, these findings reveal a novel link between apoC3 and platelet biology with potential implications for thrombotic risk and vascular homeostasis.

## 1. Introduction

Dyslipidemia remains a key modifiable risk factor for cardiovascular disease (CVD), despite significant advances in low-density lipoprotein (LDL) cholesterol-lowering therapies [1]. Although these therapies have proven effective in reducing CVD risk, a substantial residual risk persists, largely attributed to elevated triglycerides and cholesterol-rich lipoprotein remnants [2]. Apolipoprotein C3 (apoC3), a glycoprotein primarily produced by the liver and intestine, plays a pivotal role in lipid metabolism by inhibiting the clearance of triglyceride-rich lipoproteins. It exerts this effect through two main mechanisms: hindering lipoprotein lipase access to triglycerides and inhibiting hepatic uptake of lipoprotein remnants [3]. Plasma levels of apoC3 are positively correlated with circulating triglycerides and are frequently elevated in metabolic conditions such as hypertriglyceridemia and insulin resistance [3]. Previous studies have shown that apoC3 levels are associated with cardiovascular mortality [4] and the incidence of coronary artery disease [5]. A meta-analysis of 11 studies estimated that each 5 mg/dL increase in total apoC3 corresponds to a 33% rise in CVD risk [6].

Initial research posited a pro-inflammatory function for APOC3 based on its ability to stimulate the release of interleukin-1 beta from human monocytes [7,8]. However, a subsequent study demonstrated that pro-inflammatory effects are only seen when apoC3 is not lipid-bound [7,8]. Therefore, the atherogenic effects attributed to apoC3 are primarily mediated by its influence on the metabolism of triglyceride-rich lipoproteins. This mechanistic understanding highlights the importance of apoC3 as a potential therapeutic target for reducing residual cardiovascular risk [9]. Volanesorsen, an antisense oligonucleotide (ASO) targeting apoC3 mRNA, demonstrated substantial reductions in both apoC3 and triglyceride levels in the phase 3 APPROACH trial [10]. However, its clinical utility has been limited by a high incidence of thrombocytopenia (76%), necessitating careful platelet monitoring [11]. Despite this, Volanesorsen received regulatory approval for treating familial chylomicronemia syndrome under strict safety protocols [10,11]. Newer therapeutics, including Olezarsen, a next-generation ASO, and ARO-apoC3, a small interfering RNA (siRNA), have shown comparable efficacy in reducing apoC3 and triglyceride levels, with improved safety profiles [12,13]. Interestingly, in a hamster model, apoC3 deficiency exacerbated diet-induced atherosclerosis, though it improved hepatic steatosis in females [14]. Subsequent findings in apoC3-deficient animals suggest a mechanistic link between apoC3, altered platelet indices, and atherogenesis [14]. 

These results underscore the importance of further investigating the physiological role of apoC3 beyond lipid regulation, particularly its involvement in platelet biology and thrombosis. In this study, we aimed to characterize the impact of apoC3 on human platelet function and thrombus formation.

## 2. Materials and Methods

### 2.1. Materials

Alexa Fluor 488 carboxylic acid-2,3,5,6-tetrafluorophenyl ester (A-30005), intracellular (IC) fixation buffer (00-8222-49), cytochalasin B (J65342.MA), β-mercaptoethanol (21985023), iBlot3 transfer mini PVDF stacks (IB34002) and 3,3’-Dihexyloxacarbocyanine Iodide (D273) were purchased from Thermo Fisher Scientific (Vienna, Austria). Fluo-3-AM was obtained from Life Technologies (Vienna, Austria). ADP, collagen, and thrombin were purchased from Probe&Go (Osburg, Germany). Human apoC3 (isolated from human serum, P81-116), human apoA1 (P81-104), apoC3-sepharose 4B (resin coupled to a polyclonal anti-apoC3 antibody, S81-116), and rabbit anti-apoC3 primary antibody (A81-116A) were purchased from Fortis Life Sciences (Montgomery, TX, USA). FACS-Flow, Annexin V, FITC-conjugated mouse anti-human CD62P (P-selectin) antibody (550866), and FITC-conjugated mouse anti-human PAC-1 antibody (340507) were obtained from BD Bioscience (Vienna, Austria). Secondary HRP-conjugated anti-goat secondary antibody (805-035-180) was obtained from Jackson ImmunoResearch (West Grove, PA, USA). Tirofiban (SML0246) and calcium-ionophore (A23187) were purchased from Merck (Vienna, Austria). Abciximab (ab275976) was purchased from Abcam (Cambridge, UK). 

### 2.2. Immunopurification of apoC3

Immunoaffinity purification of apoC3 was performed from human sera of healthy volunteers with modifications as described [15]. Serum was incubated overnight at 4 °C with gentle rotation using Sepharose 4B resin coupled to a polyclonal anti-apoC3 antibody (S81-116) at a 1:1 ratio (1 mL of serum to 1 mL of antibody resin). The unbound fraction was collected, and the resin was then washed three times with PBS. ApoC3 was eluted three times by incubating the resin for 5 min with 3 M sodium thiocyanate. The combined eluates were concentrated using Vivaspin Turbo 15 columns (Avantor Sciences, Vienna, Austria) and buffer-exchanged into PBS using PD MiniTrap G-10 columns (Fisher Scientific, Vienna, Austria). The protein content was quantified at 280 nm using a NanoDrop microvolume spectrophotometers (Thermo Fisher, Vienna, Austria).

### 2.3. Blood Collection and Platelet Preparation

The study was conducted in accordance with the Declaration of Helsinki and approved by the Ethics Committee of the Medical University of Graz (Nr.: 21-523 ex 09/10). Participants were seemingly healthy individuals aged 30 to 45 years. Subjects who had taken nonsteroidal anti-inflammatory drugs within 24 h before blood collection were excluded. In accordance with the guidelines of the Institutional Review Board of the Medical University of Graz, all volunteers provided written informed consent. All procedures were performed in accordance with the approved protocols. Blood was collected in 3.8% (*w*/*v*) sodium citrate tubes, and platelet-rich plasma (PRP) was prepared by centrifugation at 400× *g* for 20 min at room temperature (RT), as previously described [16]. Next, 20 mL of PRP was mixed with 1 mL of 2% EDTA and centrifuged at 1000× *g* for 15 min at RT. Subsequently, platelets were washed twice with a low-pH platelet wash buffer (140 mM NaCl, 10 mM NaHCO_3_, 2.5 mM KCl, 0.9 mM Na_2_HPO_4_, 2.1 mM MgCl_2_, 22 mM C_6_H_5_Na_3_O_7_, 0.055 mM glucose monohydrate, and 0.35% bovine serum albumin, pH = 6.5) by centrifugation (1000× *g* for 15 min at RT). The final platelet preparation was resuspended in 1 mL of Tyrode’s buffer (10 mM HEPES, 134 mM NaCl, 1 mM CaCl_2_, 12 mM NaHCO_3_, 2.9 mM KCl, 0.34 mM Na_2_HPO_4_, 1 mM MgCl_2_, and 0.055 mM glucose, pH = 7.4). In our platelet aggregation experiments, we maintained a physiologically relevant concentration of platelets. After isolation and washing, the final volume of the platelet suspension was normalized to the initial plasma volume of 20 mL. This ensured consistent and functional platelet assays across all experiments. The number of platelets varied by donor, ranging from 150,000 to 250,000 per µL.

### 2.4. Platelet Aggregation

Platelet aggregation was measured at 37 °C under constant stirring (1000 rpm) using a four-channel platelet aggregometer (APACT4004, LABiTec, Ahrensburg, Germany), based on the principle of light transmission, as previously described [17]. For platelet stimulation, concentrations of ADP or collagen that elicited 70–90% aggregation were selected. Because platelet responsiveness to these agonists varies among donors, the concentrations used ranged from 3 to 10 μM for ADP and from 2.5 to 10 μg/mL for collagen in individual experiments. Platelets were preincubated for 5 min at 37 °C with vehicle, human apoC3 (1–10 μg/mL), serum, apoC3-depleted serum, or apoC3-nanoparticles (apoC3-NP). Aggregation was then induced with ADP (in the presence of 1 μg/mL fibrinogen) or collagen (1.25–10 μg/mL) and monitored for 10 min. Data at 300 s post-stimulation, corresponding to peak aggregation, were used for quantification. Aggregation is expressed as a percentage of maximum light transmission, where non-stimulated washed platelets represent 0%, and stimulated, vehicle-treated platelets represent 100%.

### 2.5. ApoC3 Binding Assay

Human apoC3 was fluorescently labeled with Alexa Fluor 488 carboxylic acid-2,3,5,6-tetrafluorophenyl ester (A-30005) according to the manufacturer’s instructions. A volume of 5 µL of washed platelets was diluted with 115 µL of Tyrode’s buffer. The platelets were stimulated with ADP (3 μM) in the presence of cytochalasin B (5 μg/mL) for 5 min at RT. Cytochalasin B was introduced to disrupt actin filaments in platelets, enabling the study of molecular pathways in the absence of confounding aggregate effects [18]. For inhibition experiments, platelets were pretreated with tirofiban (0.3–3 µM) or abciximab (1–25 µg/mL) for 15 min at RT. Afterward, the platelets were treated with apoC3 (0.1–100 µg/mL) for 30 min to allow binding. Excess apoC3 was removed by washing the platelets by centrifugation and by replacing the buffer. The platelets were fixed with IC fixation buffer (10 min, 4 °C) and analyzed by flow cytometry. 

### 2.6. Thrombus Formation Under Flow Conditions

Human platelet adhesion in whole blood to a microfluidic chip surface (Vena8 Fluoro+ biochips, 400 µm channel width, Cellix Ltd., Dublin, Ireland) was assessed on a Cellix system. The chips were coated with 250 µg/mL collagen at 4 °C overnight in a humidified box. The next day, the chips were washed twice with distilled water, blocked with 0.1 % bovine serum albumin for 30 min, and rinsed with distilled water. Whole blood from healthy donors was labeled with 20 µM 3,3-dihexyloxacarbocyanine iodide (10 µL of 10 mM stock for 5 mL of whole blood) for 10 min. After incubation with either vehicle or 10 µg/mL apoC3 for 10 min, whole blood was re-calcified by adding CaCl_2_ (1 mM final concentration) for 2 min. Then, 100 µL of blood was perfused over the collagen-precoated perfusion channel with a shear rate of 30 dynes m^−2^. Using a Hamamatsu ORCA-03G digital camera (Hamamatsu Photonics, Hamamatsu City, Japan) and Cellix VenaFlux software, thrombus formation was recorded for 3 min with a Zeiss Axiovert 40 CFL microscope with a Zeiss A-Plan 10×/0.25 Ph1 lens (Zeiss, Oberkochen, Germany). Computerized image analysis was performed using DucoCell software (Cellix, Dublin, Ireland), where the area covered by the thrombus was calculated. Data are expressed as arbitrary units (a.u.).

### 2.7. Necrotic-like and Apoptotic-like Processes

The platelets were preincubated for 15 min with either the positive control calcium-ionophore (1 µM) or human apoC3 (0.1–10 μg/mL). Calcium-ionophore induces a sudden rise in intracellular calcium, which triggers multiple platelet death-like pathways. After treatment, exposure of phosphatidylserine on the outer membrane as a marker of necrotic-like and apoptotic-like processes was evaluated using annexin V/propidium iodide staining, as previously described [19].

### 2.8. Expression of P-Selectin (CD62P)

Washed platelets, resuspended in Tyrode’s buffer, were pre-incubated with either vehicle or human apoC3 (1–10 µg/mL) for 10 min at RT. The cells were then stimulated with ADP (3 µM) in the presence of cytochalasin B (5 µg/mL) and an anti-CD62P-FITC-conjugated antibody for 30 min at RT. Cytochalasin B was added to promote the movement of P-selectin from intracellular granules to the platelet surface, as previously described [20]. The samples were fixed, and P-selectin upregulation was detected by flow cytometry.

### 2.9. GPIIb/IIIa (PAC-1) Activation

Platelet-rich plasma was suspended in Tyrode’s buffer and pretreated with vehicle or human apoC3 (1–10 µg/mL) for 10 min at RT. GPIIb/IIIa activation was induced by adding 3 µM ADP and FITC-conjugated anti-PAC-1 antibody (recognizing the activated GPIIb/IIIa conformation, clone PAC-1, Nr. 340507) and incubating for 30 min at RT. Following fixation, GPIIb/IIIa activation was assessed by flow cytometry.

### 2.10. Western Blot Analysis

Serum and apoC3-depleted serum were prepared for Western blot analysis by mixing with 6X Laemmli buffer containing 5% β-mercaptoethanol and heating at 95 °C for 5 min. Equal protein amounts were then separated by SDS-PAGE and blotted for 7 min with 20 V using the iBlot 3 system (Thermo Fisher, Vienna, Austria) with iBlot3 transfer mini PVDF stacks (IB34002). Nonspecific binding sites were blocked using a buffer containing 137 mM NaCl, 20 mM Tris, 0.1% Tween-20, and 5% milk powder (pH 7.6). The membrane was then incubated overnight at 4°C with a goat anti-apoC3 primary antibody (A81-116A) at a 1:5000 dilution. Following three washes with buffer (137 mM NaCl, 20 mM Tris, 0.1% Tween-20, pH 7.6), the membrane was incubated with an HRP-conjugated anti-goat secondary antibody (805-035-180) at a 1:5000 dilution for 1 h at room temperature. Protein bands were visualized using an iBright Imaging System (Thermo Fisher, Vienna, Austria) and ECL Blotting Substrate (Bio-Rad, Vienna, Austria). Band intensities were quantified using iBright Analysis software 5.4.0 (Thermo Fisher, Vienna, Austria).

### 2.11. Statistical Analysis

Data are presented as mean ± SEM from n independent experiments, each performed three to six times using platelets from different donors. Statistical analysis was performed using GraphPad Prism version 10. Due to the small sample size, we used non-parametric statistical tests. Two-group comparisons were made using the Mann–Whitney test. For multiple group comparisons, we used the Kruskal–Wallis test with multiple comparisons. Statistical significance was defined as * *p* < 0.05, ** *p* < 0.01, and *** *p* < 0.001.

## 3. Results

### 3.1. ApoC3 Exerts a Dose-Dependent Inhibitory Effect on Platelet Aggregation

**Figure 1:**
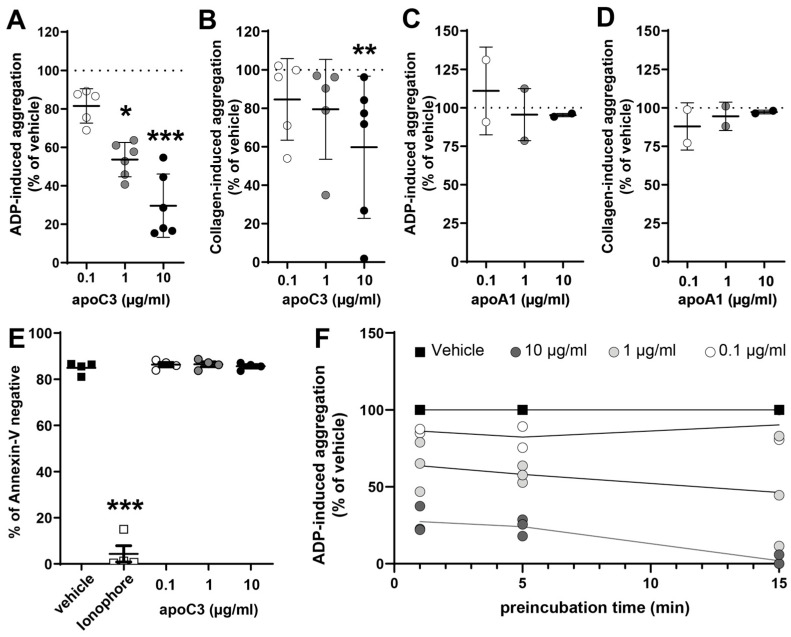
ApoC3 inhibits platelet aggregation without inducing cytotoxic-like effects. Washed platelets were preincubated with (**A**,**B**) human apoC3 (0.1–10 μg/mL) or (**B**,**C**) human apoA1 (0.1–10 μg/mL) for 5 min at 37 °C. Platelets were then stimulated with (**A**,**C**) ADP (3 to 10 μM) or (**B**,**D**) collagen (2.5–10 μg/mL) depending on the donor to achieve a platelet aggregation of ~70% for the vehicle treatment. Data at 300 s post-stimulation, corresponding to peak aggregation, were used for quantification. Results are expressed as a percentage of maximum light transmission (0% = non-stimulated platelets; 100% = vehicle-treated, stimulated platelets) (n = 2–6). (**E**) Platelets were preincubated with either human apoC3 (0.1–10 μg/mL) or the multiple platelet death-like pathway inducing agent calcium-ionophore (1 µM). Following incubation, exposure of phosphatidylserine on the outer membrane was assessed by flow cytometry using annexin V staining. Annexin V-negative cells were classified as viable, and annexin V-positive cells as procoagulant. Data are presented as a percentage of total cells (n = 4). (**F**) Platelets were preincubated with apoC3 for 1–15 min before ADP-induced aggregation (in the presence of 1 μg/mL fibrinogen). Data were analyzed using the Kruskal–Wallis test (n = 3). * *p* < 0.01; ** *p* < 0.001; *** *p* < 0.0001.

To investigate the role of apoC3 in platelet function, we performed a series of aggregation assays. We assessed platelet aggregation by light transmission aggregometry, which measures the increase in optical transmittance as platelets aggregate in response to agonists. The platelets were incubated with human apoC3 (apoC3), and aggregation was initiated using either adenosine diphosphate (ADP) or collagen as agonists. Our findings reveal a significant and dose-dependent suppression of ADP-induced platelet aggregation by apoC3 across all concentrations tested (Figure 1A). In contrast, the inhibitory effect on collagen-induced aggregation was observed only at the highest apoC3 concentration (Figure 1B). This suggests that apoC3 primarily affects ADP-mediated platelet activation pathways. To confirm the specificity of this effect, we used human apoA1 as a control [21]. ApoA1 at these concentrations had no impact on platelet aggregation (Figure 1C,D). To rule out the possibility that reduced aggregation was due to cytotoxic-like effects, we assessed platelet procoagulant activity using annexin V/propidium iodide staining. ApoC3 did not increase phosphatidylserine exposure or membrane compromise (Figure 1E). As a positive control for the apoptosis assay, we used calcium-ionophore, an agent which induces multiple platelet death-like pathways in platelets due to a sudden rise in intracellular calcium. Finally, a time-course analysis revealed that apoC3 rapidly inhibited platelet aggregation, with measurable effects seen after just 1 min of pre-incubation (Figure 1F), suggesting a fast-acting, possibly receptor-mediated mechanism.

### 3.2. Enhanced Platelet Aggregation Is Linked to ApoC3 Depletion of Serum

**Figure 2:**
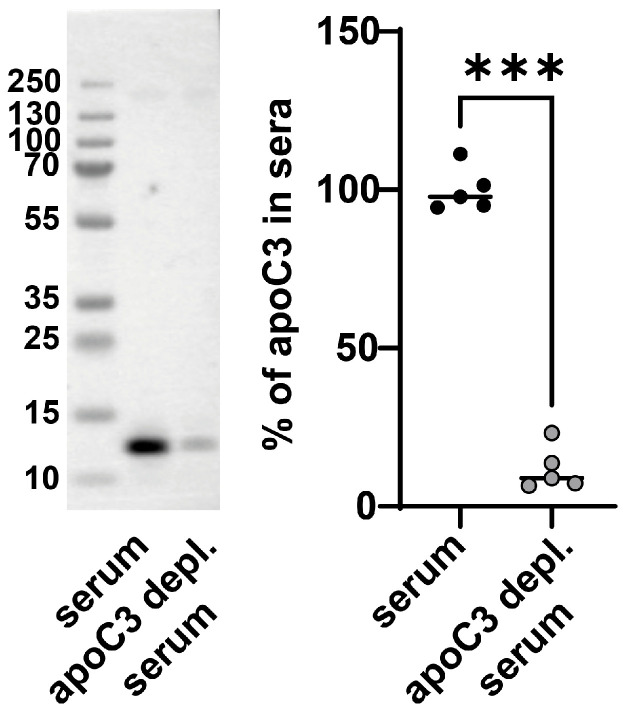
ApoC3 depletion from human serum. Quantification of apoC3 removal from human serum by immunoaffinity purification. A representative Western blot and quantification from five independent experiments are shown. Data were analyzed using the Mann–Whitney test. *** *p* < 0.0001.

**Figure 3:**
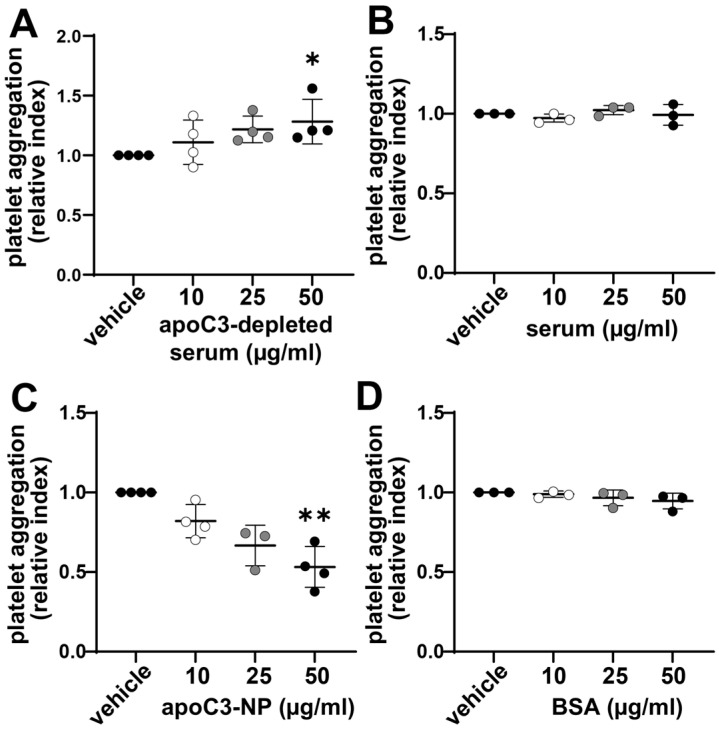
ApoC3 depletion enhances platelet aggregation in vitro. Washed platelets were preincubated for 5 min at 37°C with (**A**) apoC3-depleted serum, (**B**) full serum, (**C**) apoC3-nanoparticles (apoC3-NP), or (**D**) bovine serum albumin (BSA). Platelet aggregation was induced with ADP (3 to 10 μM) depending on the donor to achieve a platelet aggregation of ~70% for the vehicle treatment and monitored for 10 min. Data at 300 s post-stimulation, corresponding to peak aggregation, were used for quantification. Results are expressed as relative index of maximum light transmission, where 1 represents 100% of vehicle-treated aggregation of ADP-stimulated platelets. Data were analyzed using the Kruskal–Wallis test (n = 3–4). * *p* < 0.01; ** *p* < 0.001.

To study the effects of apoC3 on platelet aggregation in a physiological environment, we immunodepleted apoC3 from human serum using affinity purification. In contrast to isolated and delipidated apoC3 (used in Figure 1), the immunopurified apoC3 nanoparticles (apoC3-NP) are associated with other proteins and lipids. In the subsequent experiments, we will use the term apoC3, for isolated delipidated pure apoC3, and apoC3-NP for immunopurified apoC3-nanoparticles. Western blot analysis confirmed a robust reduction of 90% in apoC3 levels (Figure 2). When platelets were incubated with apoC3-depleted serum, we observed a dose-dependent increase in aggregation (Figure 3A). In contrast, platelets exposed to whole serum (with endogenous apoC3) exhibited no significant change in aggregation (Figure 3B). The addition of apoC3-NPs at physiologically relevant concentrations (10–50 µg/mL) [8] led to a concentration-dependent reduction in platelet aggregation, with ~50% inhibition at the highest dose (Figure 3C). Control experiments using albumin showed no effect on platelet responses (Figure 3D). These results suggest that apoC3-NPs has a constitutive inhibitory effect on platelet activity and that its removal reveals underlying proaggregatory factors in serum.

### 3.3. ApoC3 Inhibits Thrombus Formation Under Flow Conditions

**Figure 4:**
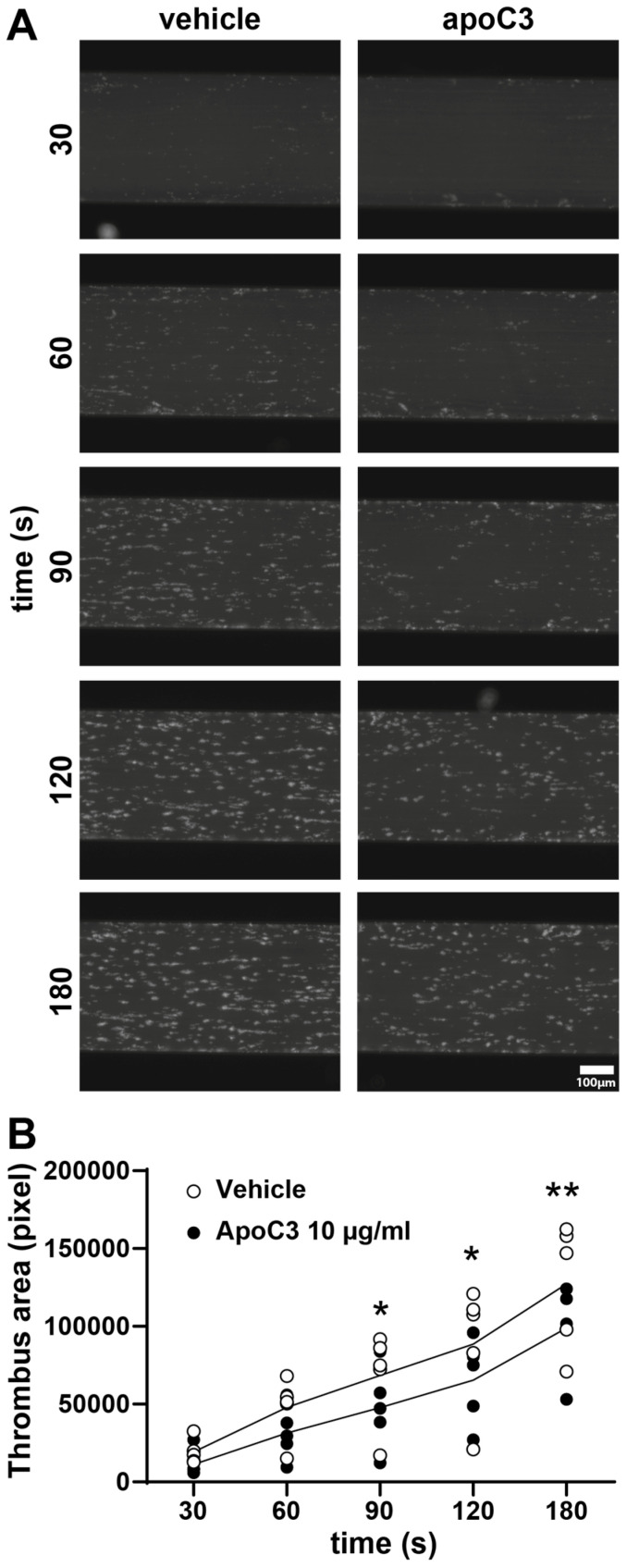
Ex vivo thrombogenesis. Whole blood was preincubated with vehicle or 10 µg/mL human apoC3 and perfused over collagen-coated channels. (**A**) Representative photomicrographs are shown for the indicated time points. (**B**) Computerized image analysis was performed using DucoCell software to calculate the thrombus-covered area. Data are expressed in arbitrary units (a.u.). The scale bar represents 100 µM. Data were analyzed using the Kruskal–Wallis test (n = 4–5). * *p* < 0.05; ** *p* < 0.01.

To evaluate the effect of human apoC3 on platelet adhesion and thrombus formation under physiologically relevant conditions, we employed a microfluidic assay using the Cellix flow system. This platform simulates in vivo hemodynamic conditions by perfusing whole human blood over a collagen-coated surface. Despite the endogenous presence of apoC3 in whole blood, supplementation with human apoC3 significantly suppressed collagen-induced thrombus formation (Figure 4A). Quantitative analysis revealed a ~25% reduction in thrombus area beginning at 90 s post-initiation (Figure 4B), indicating that apoC3 attenuates platelet-driven thrombogenesis even in the context of intact whole blood.

### 3.4. ApoC3 Reduces GPIIb/IIIa Activation and P-Selection Expression

**Figure 5:**
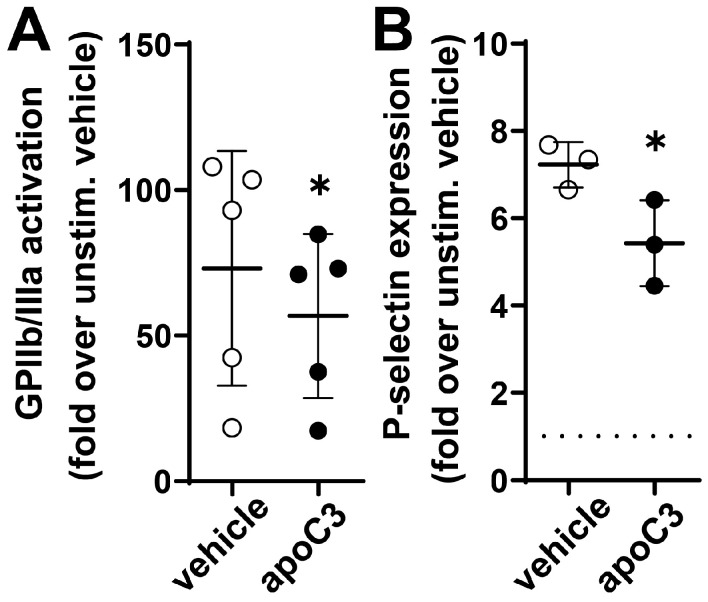
ApoC3 inhibits GPIIb/IIIa activation and P-selectin expression in platelets. Platelets were pretreated with vehicle or human apoC3 (10 μg/mL). (**A**) For GPIIb/IIIa activation, platelets were stimulated with ADP (3 μM). (**B**) Surface expression of P-selectin was induced with ADP (3 μM) in the presence of cytochalasin B (5 μg/mL). (**A**,**B**) GPIIb/IIIa activation and P-selectin expression were measured by flow cytometry. Data are expressed as fold over unstimulated (without ADP) control from mean fluorescence values. Results are shown as mean ± SEM (n = 3–5). Data were analyzed using the Mann–Whitney test. * *p* < 0.05.

To elucidate the molecular mechanisms by which apoC3 inhibits platelet activation, we examined the process of GPIIb/IIIa activation and the subsequent surface expression of P-selectin, two well-established markers of platelet activation. GPIIb/IIIa, the platelet fibrinogen receptor, undergoes a conformational change upon ADP stimulation, enabling fibrinogen binding and promoting platelet aggregation. P-selectin, a membrane protein stored in alpha-granules, is translocated to the platelet surface during later stages of activation. It serves as an indicator of granule release and sustained platelet activation [22,23]. Treatment with human apoC3 led to a significant reduction in ADP-induced GPIIb/IIIa activation, suggesting interference with integrin activation (Figure 5A). Furthermore, apoC3 significantly reduced P-selectin expression on platelet surfaces (Figure 5B), indicating suppression of platelet degranulation and activation. These findings support a role for apoC3 as a direct negative regulator of platelet signaling pathways central to thrombus development.

### 3.5. ApoC3 Directly Interacts with Glycoprotein IIb/IIIa (GPIIb/IIIa) 

**Figure 6:**
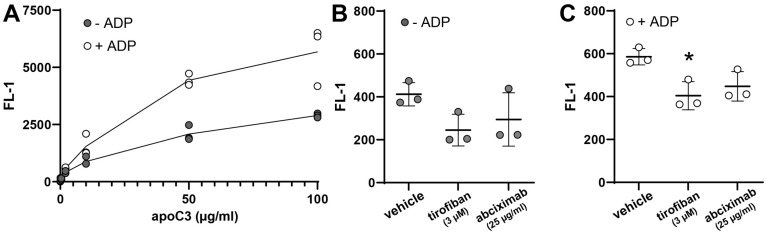
ApoC3 binding to platelets. (**A**) Platelets were stimulated with 3 µM ADP for 5 min at room temperature (RT). Then, 0.1–100 µg/mL human apoC3-FITC was added, and after a 30-min incubation to allow binding, excess apoC3-FITC was removed. Platelets were fixed and analyzed by flow cytometry (n = 3). For inhibition experiments, platelets were pretreated with (**B**) tirofiban (3 μM) or (**C**) abciximab (25 μg/mL) for 15 min at RT in the absence or presence of ADP before the addition of apoC3 (2 μg/mL). Data were analyzed using the Kruskal–Wallis test. * *p* < 0.05.

To identify the binding proteins through which apoC3 modulates platelet function, we first examined its direct binding to platelets. Using flow cytometry and fluorescently labeled apoC3, we observed a dose-dependent increase in binding to resting platelets (Figure 6A). Interestingly, pre-activation of platelets with ADP markedly enhanced apoC3 binding, suggesting that platelet activation increases receptor accessibility. Given the central role of GPIIb/IIIa in platelet activation and aggregation, we hypothesized that GPIIb/IIIa may serve as a direct binding site for apoC3. Furthermore, as illustrated in Figure 5A, treatment with apoC3 significantly reduced ADP-induced GPIIb/IIIa activation, indicating direct binding. To directly test this, we assessed apoC3 binding in the presence of two GPIIb/IIIa antagonists, tirofiban and abciximab, which both inhibit fibrinogen binding but differ in binding properties: tirofiban is a reversible inhibitor, whereas abciximab binds with high affinity and acts effectively irreversibly. Tirofiban reduced apoC3 binding under both resting (*p* = 0.07) and ADP-stimulated conditions (*p* = 0.04) (Figure 6B,C), demonstrating that apoC3 binding is at least partially dependent on GPIIb/IIIa. Abciximab also attenuated apoC3 binding in ADP-activated platelets (Figure 6C), although not statistically significant (*p* = 0.22). These findings identify GPIIb/IIIa as a principal receptor for apoC3 in activated platelets, establishing a direct molecular link between apoC3 and this integrin, which is central to platelet aggregation. Notably, the partial inhibition observed with both antagonists suggests the presence of additional apoC3-binding sites on platelets.

## 4. Discussion

Our study reveals an unrecognized role of apoC3 in negatively regulating platelet activation and thrombus formation. Using a combination of depletion and reconstitution approaches, we demonstrate that removing apoC3 from human serum enhances platelet aggregation. Conversely, supplementation with human apoC3 or apoC3-NPs significantly inhibits aggregation. Our findings reveal a significant and dose-dependent suppression of ADP-induced platelet aggregation by apoC3 across all concentrations tested, whereas the inhibitory effect on collagen-induced aggregation was observed only at the highest apoC3 concentration. ADP-dependent aggregation is primarily mediated via the P2Y12 receptor, triggering inside-out signaling that activates integrins. In contrast, collagen-induced aggregation engages GPVI, which is coupled to the FcRγ chain and a robust tyrosine kinase signaling cascade, leading to stronger inside-out activation of integrins and subsequent platelet responses. Notably, the collagen–GPVI pathway is generally more potent than ADP in driving platelet activation, reflecting its ability to induce stronger calcium fluxes, granule secretion, and integrin activation [24]. Despite these differences in upstream signaling, both ADP- and collagen-dependent aggregation converge on the outside-in signaling of GPIIb/IIIa, which mediates fibrinogen binding, platelet spreading, and stable aggregation. 

Under flow conditions, apoC3 reduced thrombus formation, indicating a broader role in hemostatic regulation. ApoC3 attenuated key markers of platelet activation, including GPIIb/IIIa activation and P-selectin surface expression. Mechanistically, apoC3 binding to platelets increased with activation and was partially mediated by GPIIb/IIIa, implicating this integrin as a functionally relevant receptor. While our data show that GPIIb/IIIa antagonists (tirofiban and abciximab) partially reduce apoC3 binding, we acknowledge that some of this reduction may result from the general inhibitory effect of these antagonists on platelet activation. Taken together, these findings reveal a novel link between apoC3 and platelet biology, with potential implications for thrombotic risk and vascular homeostasis.

Currently available lipid-lowering therapies, including fibrates, fish oil, niacin, statins, and ezetimibe, modestly reduce apoC3 levels by 10–30% [25]. In contrast, newer antisense oligonucleotide and siRNA-based therapies targeting apoC3 have demonstrated far greater efficacy in lowering both apoC3 and triglyceride levels, with several agents (Olezarsen and Plozasiran) now in late-stage clinical trials [10,12]. Olezarsen, an N-acetylgalactosamine-conjugated antisense oligonucleotide, has been shown to lower apoC3 by an average of 68% [26]. Volanesorsen, tested in patients with hypertriglyceridemia and type 2 diabetes, achieved even greater reductions of up to 88% [27]. Importantly, no significant thrombocytopenia has been reported with Olezarsen, whereas Volanesorsen has been associated with dose-dependent thrombocytopenia, necessitating close monitoring and treatment adjustments [10,11]. While the present study focused on the direct effects of apoC3 on platelet activation and aggregation, future investigations should assess whether pharmacological lowering of apoC3 by these agents exerts similar effects on platelet biology. In our experiments, the extent of apoC3 suppression was comparable to, or greater than, that achieved with Volanesorsen, raising the possibility that profound reductions in apoC3 may contribute mechanistically to platelet activation and, ultimately, thrombocytopenia. Additional studies will be necessary to determine whether alternative strategies can effectively lower apoC3 while preserving platelet safety. Normal plasma apoC3 concentrations typically range from approximately 8 to 12 mg/dL, with mean levels around 12.5 mg/dL in the general population [28]. The maximum concentration tested in our aggregation studies (10 μg/mL = 1 mg/dL) is approximately 10-fold lower than these physiological levels. However, it is important to note that the lipidation status of apoC3 significantly influences its biological activity. ApoC3 is present in various lipoprotein particles, including triglyceride-rich lipoproteins (TRLs) such as chylomicrons and very-low-density lipoproteins, as well as high-density lipoproteins. The functional properties of apoC3 can vary depending on its association with these lipoproteins. Platelet surface receptors like GPIIb/IIIa (~10–15 nm in extracellular domain) are much smaller and localized in tight spaces, such as the platelet membrane and filopodia. The large size of TRLs may sterically hinder direct access of apoC3 embedded in the TRL surface to platelet receptors. ApoC3 in TRLs is largely associated with the lipoprotein surface, possibly buried among other apolipoproteins and phospholipids, which could reduce its effective accessibility to platelet receptors. In contrast, purified apoC3 used in vitro is fully exposed and able to interact directly with platelet integrins. Therefore, the inhibitory effects observed in vitro with purified apoC3 may not fully translate to the same effect in vivo under normal lipoprotein-bound conditions. 

Our findings appear to diverge from recent reports that frame apoC3 as a pro-thrombotic and pro-inflammatory molecule. Clinical studies have associated elevated apoC3 levels with cardiovascular mortality and increased thrombin generation [29]. Experimental models further suggest that apoC3, particularly when carried on TRLs, promotes reactive oxygen species (ROS) production and downstream vascular inflammation [30]. The apparent discrepancy may reflect differences in experimental context. Our work examines the acute effects of purified apoC3 on healthy platelets in vitro, while prior studies often capture the consequences of chronic exposure in dyslipidemic states in vivo. We propose that apoC3 may exert dual effects: (i) an immediate, receptor-mediated inhibitory action on platelet activation, and (ii) a long-term pro-inflammatory influence mediated by inhibition of TLR clearance and subsequent TRL-associated ROS. An alternative but complementary hypothesis, supported by Li et al. and Hosseini et al., is that chronic apoC3-driven ROS generation may induce “platelet exhaustion” [30,31]. This process involves calpain-mediated cleavage of the integrin β3 subunit, impairing platelet function and contributing to long-term vascular pathology [31]. Future studies assessing ROS dynamics under acute and chronic conditions will be critical to reconcile these dual roles.

Our identification of GPIIb/IIIa as a binding partner for apoC3 underscores the integrin’s central role in platelet biology. Beyond serving as a fibrinogen receptor, GPIIb/IIIa functions as a signaling hub: inside-out signals increase ligand affinity, while subsequent ligand binding triggers outside-in signaling, leading to granule release, cytoskeletal remodeling, and platelet spreading [31]. We found that apoC3 reduced both GPIIb/IIIa activation (PAC-1 binding) and P-selectin expression, suggesting that its binding interferes with this entire signaling cascade. One plausible mechanism is that apoC3 locks GPIIb/IIIa in a conformation unable to support high-affinity ligand binding or downstream signaling, thereby exerting broad inhibitory effects on platelet function.

Interestingly, we observed that GPIIb/IIIa antagonists only partially blocked apoC3 binding, suggesting the involvement of additional binding sites. While apoA1 served as a negative control and showed no effect in the concentrations tested in our assays, the broader literature indicates that several apolipoproteins can engage specific platelet receptors and influence platelet activity [32]. Identifying other apoC3-binding sites on platelets therefore represents an important area for future research.

While the triglyceride-lowering effects of apoC3 inhibition are well established, its impact on atherosclerotic cardiovascular disease remains less clear. ApoC3 deficiency lowers plasma triglycerides and cholesterol while raising HDL-C levels, suggesting a potentially atheroprotective lipoprotein profile [33]. Mendelian randomization studies support a causal relationship between apoC3 loss of function variants and reduced cardiovascular risk, particularly in European populations [34,35]. However, this association appears to be population-specific. For example, although certain rare variants (e.g., rs138326449) confer cardioprotection in Europeans, similar benefits have not been consistently observed in Asian Indian cohorts, despite significant triglyceride reductions [34,35]. Furthermore, the role of apoC3 in atherogenesis is not unequivocally beneficial. The lack of significant impact on lipid metabolism and atherogenesis observed in LDLR-/- mice lacking apoC3 suggests that targeting apoC3 for the treatment of hyperlipidemia and CVD may require further investigation [30]. Moreover, apoC3 deletion in LDLR^−/−^ hamsters paradoxically promoted atherosclerosis despite improvements in triglyceride levels and hepatic lipid markers [14]. In this model, ApoC3 deficiency was associated with altered platelet indices, which is consistent with our findings suggesting that apoC3 modulates platelet activity. However, human heterozygous carriers of inactivating apoC3 mutations exhibited lower triglyceride and apoC3 levels without alterations in platelet count [36], suggesting that a partial reduction in apoC3 may preserve its antithrombotic function. 

It is plausible that other apolipoproteins may affect platelet activity in a similar manner. For instance, apoA4 has been shown to reduce platelet aggregation by antagonizing GPIIb/IIIa [32], which is analogous to the inhibitory effects of apoC3 that we observed. Interestingly, the postprandial rise in apoA4 is believed to mitigate lipid-induced platelet hyperactivity and vascular inflammation [32]. Taken together, these results imply that apoC3 and apoA4 may have evolved as complementary regulators of hemostasis and inflammation. These mechanisms could have developed to counteract the prothrombotic and proinflammatory effects of the intermittent, high-fat meals that were characteristic of the original human diet. We acknowledge some limitations of this study. First, experiments were conducted using in vitro assays, such as light transmission aggregometry and thrombus formation under flow. These assays do not fully capture the complexity of the in vivo hemostatic environment. Nevertheless, using human platelet-rich plasma, human native sera, and whole blood enhances the physiological relevance of our findings and provides mechanistic insights.

## 5. Conclusions

In conclusion, our study reveals a novel antithrombotic function of apoC3 that expands its role beyond triglyceride metabolism to include the modulation of platelet activation and thrombosis. Our observations underscore the importance of thoroughly evaluating the implications for hemostasis and thrombotic risk in clinical development.

## Data Availability

The data that support the findings of this study are available from the corresponding author upon reasonable request (Medical University of Graz, gunther.marsche@medunigraz.at).

## Abbreviations

The following abbreviations are used in this manuscript:

ADP	Adenosine diphosphate
apo	Apolipoprotein
ASO	Antisense oligonucleotides
CVD	Cardiovascular disease
GPIIb/IIIa	Glycoprotein type IIb/IIIa
LDLR	Low-density lipoprotein receptor
PRP	Platelet-rich plasma
siRNA	Small-interfering RNA
